# Report of the Committee on Hygiene of the Medical Society of the State of New York1Presented at the ninety-fifth annual meeting at Albany, January 29, 1901.

**Published:** 1901-03

**Authors:** Henry R. Hopkins, M. A. Veeder, H. L. Elsner, John H. Pryor, J. M. Mosher, George B. Fowler

**Affiliations:** Chairman


### State Medicine.

### REPORT OF THE COMMITTEE ON HYGIENE OF THE MEDICAL SOCIETY OF THE STATE OF NEW YORK.[1]

*To the Medical Society of the State of New York :*

YOUR committee on hygiene respectfully report :

To those especially interested in the subject of hygiene the events of the year last past afford substantial reasons for encouragement.

At our last meeting the topic of hygiene was prominently before this society in addresses, communications and discussions. During the year important hearings were held before legislative committees. The senate passed a bill appropriating $150,000 for the establishment of a State Sanatorium for the treatment of incipient consumptives, and although the opposition in the lower house cut this appropriation to $50,000, they were unable to prevent the passage of the bill, which went to the governor and was signed, thus enabling the closing year of the century to witness a sociological event of the first magnitude—the State of New York taking its first step in the great cause of state prevention of consumption.

Time and opportunity to hear and weigh objections to the proposition that the state should lead in the cure and prevention of this communicable disease, has but confirmed your committee upon the importance of the subject—the wisdom and timeliness of the action heretofore taken by this society, and the committee with all emphasis commends the principle to your fostering care, and once more recommends the necessary legislation.

The attention of your committee has been directed to another topic in local hygiene—closely related to the great question of consumption and perhaps only second to it in order of importance—the housing of the poor, particularly the poor of our chief city, the metropolis of all the Americas. In the City of New York this question of the unhygienic housing of the poor working man, has been the nightmare of sanitarians for more than half a century. How practical and important this question is, we get an idea when we recall that two-thirds of the people of the city live in tenement houses, many of these without adequate air or light or space or possibility of cleanliness; live under conditions so unnatural, so unhygienic that

1. Presented at the ninety-fifth annual meeting at Albany, January 29, 1901.

disease, poverty and crime, in an alarming degree are the inevitable incidents of life. Your committee commends this matter of work for the better housing of the poor, as a subject worthy of your warm and active sympathy and support, and with greater particularity, because in this work, as in many of the instances of state medicine, prevention is so much easier, so much cheaper, so much more economical than cure. Your committee advises that restrictive ordinances as to the construction of buildings intended for the occupancy of more than three families, be enacted and enforced by every municipality in the state; also, that local health authorities be encouraged in more careful inspection and more fearless condemnation of those now known or believed to be a menace to our public health.

The State of New York annually expends some $25,000,000 in its various charities, and the greater part of this enormous sum is a penalty for our failing to protect the public health. Many think this is a speculative, a philosophic proposition; there was never a more intensely and exclusively practical proposition.

Your committee has also given consideration to the significance of the evil influence upon the public health of the two communicable diseases, gonorrhea and syphilis, and advises that efficient steps be taken to protect our people from these diseases, by bringing to bear upon them the principle of isolation, a principle of well-proven effectiveness in the control of communicable disease, from the days of Moses and the health ordinances relating to leprosy, down to the placard of today showing that there is diphtheria in the house.

To this end your committee invites consideration of the proposition: that in view of the continued existence of the communicable diseases, gonorrhea and syphilis, of the great influence of the former in the production of blindness, particularly in the newly born, and of sterility in our young men, and of pelvic disease and sterility in our young women; of the importance of the latter as a many-sided and wide-reaching factor in working individual family and racial degeneration; that it is desirable that these diseases be included in the list of communicable diseases, requiring notification and registry; that cities having boards of health take steps to secure the registry of all persons having the diseases named, and that such registration be lodged with the health authorities.

The intelligent conviction of the medical profession as to the relation of typhoid fever and impure water supply is bearing fruit. The cities of Albany and Schenectady, acting under judicious advice.

have taken steps to replace an infected water supply with pure water, with the prompt and usual result of immediate and emphatic fall in the typhoid morbility and mortality. These cities need no more to be classed with those of high typhoid prevalence, like Alexandria and Cairo, but with those of very low prevalence, like Munich and Vienna.

The result in Schenectady seems most satisfactory and impressive. Previous to 1897, that city had the unenviable notoriety of the highest typhoid mortality of any city in the state,—a state of many cities with discreditable typhoid records,—but since stopping the use of infected water the years 1898 and 1899 have passed with the lowest typhoid mortality of any of the twenty chief cities of the state. This would seem a favorable occasion to invite your attention to the scandalously high typhoid death-rate of the entire state as compared with that of many of the great cities of Europe—cities blessed with greater civic enlightenment, purer water supply and comparative immunity from typhoid morbility and mortality.

The cities of Munich, Vienna, Hague, Berlin, Rotterdam, Breslau, Hamburg, Zurich, Amsterdam, London, Edinburgh and Warsaw, are supplied with pure water and have an average annual typhoid rate of less than 8 per 100,000 population.

The cites of New York, Yonkers, Kingston, Utica, Auburn and Rochester are those of this state having the better water supply and the lower typhoid mortality, the average of the ten years preceding and including 1899, being 20.66 per 100,000 population.

While Oswego, Buffalo, Syracuse, Newburgh, Poughkeepsie, Amsterdam, Binghampton, Troy, Elmira, Watertown, Niagara Falls, Albany, Cohoes and Schenectady, use or have used an infected water supply, and during the ten years preceding and including 1899, have had an average annual typhoid mortality of 53.71 per 100,000 population; giving a total of twenty New York cities with an average typhoid mortality of 37.18, as compared with twelve cities of Europe with a typhoid mortality of 8.

Here, perhaps, we may profitably remind ourselves that this is the Medical Society of the State of New York; that this society should represent the medical profession of the state, and that in sanatory matters the medical profession is the light of the civic government, the inspiration of the civic conscience.

May we not resolve that while the new century is yet young, the deliverance from needless typhoid fever, which has been brought to the dwellers at Albany and Schenectady shall in like manner by our unceasing diligence be bestowed upon every inhabitant of the Empire State ?

And, further, that it is sometimes desirable to consider our unworthiness and that we go down into the valley of humiliation and look upon these tell-tale statistics of European and New York typhoid mortality and see them as they are, a conclusive demonstration of our professional inefficiency in preventive medicine, as compared with our more fortunate brethren of Europe; that we ask ourselves why we have permitted and still permit this enormous typhoid death-rate, this frightful loss of time, of energy, of money, of life, by the disease, of all the communicable diseases, the most easy of prevention? Why we are in sanatory efficiency, in preventive medicine, so far below and behind our brethren of Europe? And your Committee on Hygiene in making answer would remind you that the energy of the gun is never more than the potential energy of the powder burned in the discharge—that the profession of Europe brings to the attack of sanatory problems breach loading rifles and smokeless powder, while we of the State of New York are but just beginning to see the first fruits of our long and discouraging crusade against the too long standing habit of infecting the medical profession itself, not with typhoid water or tuberculous milk, but with the more deadly infection, with the ignorant graduate of the incompetent medical college.

The sanatory problems of today will be mastered only by the use of modern weapons, in the hands of men of the highest efficiency and competency; bows and arrows, totems and medals are of the past, not of the present century.

Again, the medical profession of Europe speaks with a single voice while the people of the State of New York, where they should be encouraged, enlightened and led, are confused, disheartened and angered by a babel of voices from physicians, homeopathic physicians, electic physicians, and association physicians, with osteopaths, and Eddyites expected tomorrow.

To return to our military figure, the sanatory campaigns of the Empire State of the 20th Century will be efficiently, creditably, satisfactorily conducted only by a medical profession, kept at the highest state of efficiency and competency by state examination and state license, and united, at least for the purpose of promoting the public health, in the Medical Society in the State of New York.

There will be reason for thankfulness and congratulation when the time shall come for this unification of the medical profession, if the organic law of this society and of the county societies in affiliation therewith, shall be found as now, of that simple, broad and catholic character, under which honest and positive men united by many common beliefs and aspirations, can agree upon essentials while they disagree upon that which is less essential or non-essential.

In conclusion, your committee may be permitted to observe that many signs indicate that the demands of the present century upon the science and the art of the hygienist, are likely to be more trying, more exacting than those of the last. Competent statisticians confidently predict that before this century closes, the population of this country will exceed four hundred millions of souls, hence our successors in the Medical Society of the State of New York will be responsible for the lives and health of a people nearly as numerous as the present population of the nation. History has demonstrated over and over again that hygienic problems increase in difficulty and perplexity as populations become more dense,—as nations grow older, as wealth and luxury increase. History also abundantly attests the seeming paradox, that those medical men are the more prosperous, the more blest, who live and work amidst a people whose health does most abound; that the prevention of disease wins for the physician his highest emoluments—his chiefest honors.

The men to do this work must be men equipped with high personal and professional attainments, inspired by high ideals of duty and responsibility.

Respectfully submitted,

HENRY R. HOPKINS, *Chairman.*
M. A. VEEDER, JOHN H. PRYOR,
H. L. ELSNER, J. M. MOSHER,
GEORGE B. FOWLER.

